# Association between anthropometric indices and chronic kidney disease: Insights from NHANES 2009–2018

**DOI:** 10.1371/journal.pone.0311547

**Published:** 2025-02-14

**Authors:** Xinyun Chen, Zheng Wu, Xingyu Hou, Wenhui Yu, Chang Gao, Shenju Gou, Ping Fu

**Affiliations:** 1 Department of Nephrology, Kidney Research Institutes, West China Hospital, Sichuan University, Chengdu, China; 2 Business School, Sichuan University, Chengdu, China; 3 School of Economics and Management, North China Electric Power University, Beijing, China; City College of New York, UNITED STATES OF AMERICA

## Abstract

**Introduction:**

The strong association between obesity and chronic kidney disease (CKD) has been empirically validated, yet traditional measures like the Body Mass Index (BMI) fail to accurately assess the extent of obesity due to CKD’s characteristics, such as reduced muscle mass and increased visceral fat. This study investigates the association between CKD and several anthropometric indices, including A Body Shape Index (ABSI), Body Roundness Index (BRI), Waist-to-Height Ratio (WHtR), and the Conicity Index (C-index), to determine their predictive capabilities.

**Methods:**

Based on the datasets from the National Health and Nutrition Examination Survey (NHANES) 2009–2018, weighted multivariable regression analyses were carried out to examine the independent relationship between two anthropometric indices and CKD. Also, subgroup analyses, restricted cubic spline regression (RCS), and receiver operating characteristic curve analysis were conducted for further data analyses.

**Results:**

A total of 24,162 participants were enrolled in this study. After adjusting for confounding factors, ABSI, BRI, WHtR, and the C-index were significantly associated with an increased risk of CKD, while BMI was not. Height showed a protective effect against CKD. ABSI and the C-index demonstrated the highest areas under the curve (AUCs), indicating superior predictive capabilities compared to traditional measures like BMI and waist circumference (WC). Subgroup analyses revealed significant interactions between the anthropometric indices and factors such as age, disease status, dietary intake, and physical activity levels.

**Conclusions:**

This study highlights the significant associations between various anthropometric indices (including ABSI, BRI, WHtR, and C-index) and the risk of CKD. ABSI and the C-index demonstrated the strongest predictive capabilities for CKD, with the highest AUC values.

## 1. Introduction

Obesity poses a significant and escalating global health concern, with its increasing prevalence and its association with various complications like chronic kidney disease (CKD) [1, 2]. In the United States, the reported incidence rate of obesity in adults was 39.6% [3]. Meanwhile, the prevalence of CKD has recently surged, reaching 7.5% among US adults [4]. The prevalence of obesity within the CKD population demonstrated an upward trend, with 44% of CKD patients reported to have obesity [5, 6]. A comprehensive and collaborative meta-analysis encompassing over five million individuals reported that excessive adiposity was an independent risk factor for glomerular filtration rate (GFR) decline and mortality [7]. Obesity heightens the risk of kidney disease through a multitude of mechanisms, involving insulin resistance, lipotoxicity, dysregulation of adipocytokines, increased blood pressure, and augmented glomerular blood pressure [8, 9]. Also, interventions targeting weight loss exhibit therapeutic efficacy for CKD. The weight loss achieved by behavioral modification or medications demonstrated a capacity to reduce albuminuria and, in specific instances, decelerate the rate of decline in the estimated glomerular filtration rate (eGFR) [2].

Given the reversible and preventable nature of obesity, it becomes crucial to pinpoint obesity indicators associated with the kidney function of CKD patients, which would facilitate the early detection and diagnosis of potential CKD populations in clinical practice. However, when assessing the extent of obesity in individuals with CKD, there is typically a lack of suitable indicators. Patients with CKD often manifest an altered body composition, marked by reduced muscle mass and increased visceral adiposity [2]. While the body mass index (BMI) is widely utilized in clinical practice for obesity assessment, its predictive accuracy in CKD patients is limited [10]. Certain studies even had reported a negative correlation between obesity, defined as having a BMI greater than 25, and improved survival in CKD patients, and this phenomenon was commonly referred to as the “obesity paradox” [11, 12]. Subsequent studies speculated that BMI did not consider the prevalent muscle atrophy observed in CKD patients [13, 14]. A study utilizing the Body Composition Monitor revealed that a significant proportion of individuals exhibited excess body fat despite having a normal BMI [15]. The assessment of fat mass and its distribution can also be conducted through imaging techniques including computed tomography, magnetic resonance imaging, ultrasound, and dual-energy X-ray absorptiometry (DXA) [7]. However, the application of these methods in both clinical practice and research may be constrained by the limited availability of expensive instruments, high maintenance costs, and the requirement for skilled operators. In light of this, our study aims to identify a more precise, non-invasive, and convenient indicator for evaluating the degree of obesity in CKD patients.

In this context, our focus has turned to several anthropometric indices, namely A Body Shape Index (ABSI), Body Roundness Index (BRI), Waist-to-Height Ratio (WHtR), and Conicity Index (C-index). ABSI was empirically derived by adjusting waist circumference (WC) for height and weight [16]. BRI estimates the percentage of body fat and the roundness of the body using measurements of WC and height [17]. We also employed WHtR, a simple ratio that compares an individual’s WC to their height [18], and C-index, which measures abdominal obesity by analyzing the relationship between WC, weight, and height [19]. ABSI and C-Index focus on central and abdominal obesity, respectively, providing detailed measures of fat distribution and associated health risks. BRI gives a broader perspective on body composition by estimating body fat percentage and roundness. WHtR is a straightforward measure highlighting central obesity and is useful for general health assessments. However, the relationship between these indices and CKD remains unclear, and it is still unknown which index has the better diagnostic performance for CKD. In this study, we investigated the association between different anthropometric indices with CKD in the American population, utilizing weighted data obtained from the National Health and Nutrition Examination Survey (NHANES) spanning the years 2009 to 2018. In addition to studying the relationship between ABSI, BRI, WHtR, and the C-index with CKD, we also examined the relationship between CKD and commonly used indices such as height, weight, WC, and BMI. The comparative analysis of the diagnostic performance of all these indices for CKD is finally conducted based on the area under the receiver operating characteristic (ROC) curve.

## 2. Materials and methods

### 2.1 Study population

NHANES is a comprehensive series of cross-sectional surveys that encompass the non-institutionalized civilian population of the United States (https://www.cdc.gov/nchs/nhanes/). NHANES incorporates a wide array of data, including demographic, socioeconomic, dietary, and health-related information collected through face-to-face interviews, physical and physiological examinations, as well as thorough laboratory tests.

This research analyzed de-identified information downloaded from the National Health and Nutrition Examination Survey public database. The National Center for Health Statistics Ethics Review Committee granted ethics approval. All methods were carried out in accordance with relevant guidelines and regulations (declaration of Helsinki). All individuals provided written informed consent before participating in the study.

**Fig 1:**
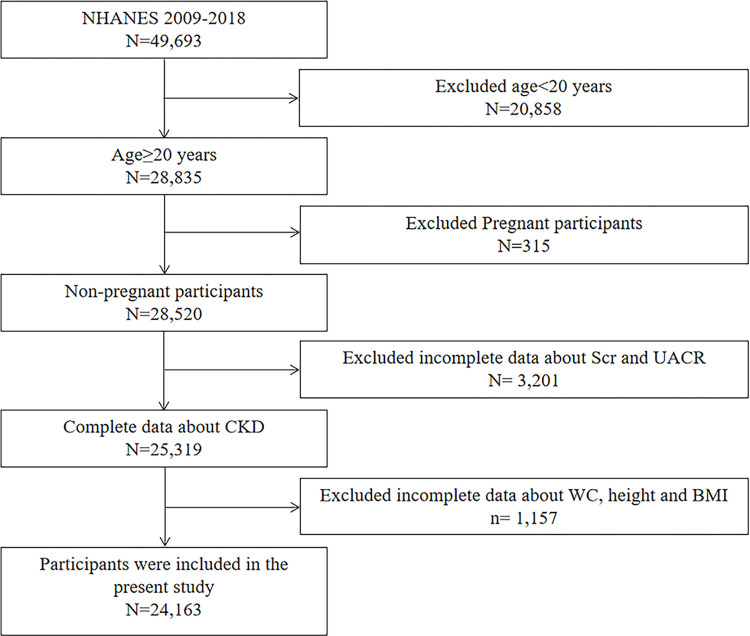
A flowchart showing the selection of study participants.

A total of 49,693 participants completed the survey across five NHANES cycles (NHANES 2009–2010, 2011–2012, 2013–2014, 2015–2016, and 2017–2018 cycles). Subsequently, we excluded 20,858 participants under the age of 20, and an additional 315 participants who were pregnant. Moreover, data on serum creatinine or urinary albumin/creatinine ratio (UACR) was not available for 3,201 participants. Data pertaining to WC, height, or BMI were lacking for 1,157 participants. Therefore, a total of 24,162 participants was included in this study (Fig 1).

### 2.2 Data collection

#### 2.2.1 Exposure variable

All parameters were assessed using standardized methods. The anthropometric indices (ABSI, BRI, WHtR and C-index) were calculated using the following equations [16, 17, 19–21]:

$$ABSI=\frac{WC}{{BMI}^{2/3}\times {height}^{1/2}}$$

$$BRI=364.2-365.5\times \sqrt{1-\frac{{\left(WC/2\pi \right)}^{2}}{{\left(0.5\times height\right)}^{2}}}$$

$$WHtR=\frac{WC}{height}$$

$$C-index=\frac{WC}{0.109\times \sqrt{\frac{weight}{height}}}$$

To enable comparability and to account for the pronounced sex differences, all anthropometric indices on a continuous scale were transformed to sex-specific z-scores.

$$z-scores=\frac{x-\mu }{\sigma }$$

*x* is the individual’s value for the anthropometric index. μ is the mean of that index for the individual’s sex group. σ is the standard deviation of that index for the individual’s sex group.

#### 2.2.2 Outcome variable

CKD is defined by a persistent abnormality in kidney structure or function for three or more months, signified by the presence of either low eGFR (below 60 mL/min/1.73 m^2^) or albuminuria (UACR equal to or exceeding 30 mg/g) [22, 23]. The calculation of eGFR was performed using the Chronic Kidney Disease Epidemiology Collaboration study (CKD-EPI) equation based on serum creatinine levels [24]. Urinary albumin was quantified using a solid-phase fluorescence immunoassay, and urinary creatinine was measured employing an enzymatic method [25].

#### 2.2.3 Assessment of covariates and outcomes

Information on covariates was predominantly gathered from three distinct sources: questionnaires, physical examinations, and laboratory data. The questionnaires encompassed inquiries regarding age, gender, race, education level, marital status, household income, smoking status, alcohol consumption, physical activities, caloric intake, and self-reported baseline medical history, including diabetes mellitus (DM), hypertension, and cardiovascular disease (CVD). Physical examinations comprised of measurements such as WC, weight, height, BMI, blood pressure. Laboratory data involved variables such as hemoglobin (HGB), albumin (ALB), total cholesterol (TC), triglyceride (TG), low-density lipoprotein (LDL), high-density lipoprotein (HDL), hemoglobin A1c (HbA1c), serum creatinine, blood urea nitrogen (BUN), UACR.

Race was classified into five groups: Mexican American, other Hispanic, non-Hispanic white, non-Hispanic black, and other. Marital status was segmented into six categories: married, widowed, divorced, separated, never married, and living with a partner. Education level was stratified into three categories: less than high school, high school, and more than high school. Family income was delineated as ≤130% (reference group), >130–350%, and >350% based on the ratio of family income to poverty (FPL) [26]. Participants with a self-reported history of congestive heart failure, coronary heart disease, angina/angina pectoris, and heart attack were diagnosed with CVD. Hypertension was defined if participants had systolic blood pressure ≥130 mmHg or diastolic blood pressure ≥90 mmHg, a physician diagnosis of hypertension, or were using antihypertensive medication. For consistency with the diagnostic criteria for DM, an expanded diagnostic cluster was utilized, incorporating six reference definitions: physician diagnosis of DM, use of antidiabetic medication or insulin, fasting glucose ≥126 mg/dL, 2-hour blood glucose ≥11.1 mmol/L during an oral glucose tolerance test, or HbA1c ≥6.5%. Alcohol consumption was dichotomized as never (having consumed <12 drinks in a lifetime), low-to-moderate (≤1 drink per day for women or ≤2 drinks per day for men on average over the past year), and severe (>1 drink per day for women or >2 drinks per day for men on average over the past year). Smoking status was categorized into three groups: never smokers (smoked <100 cigarettes in a lifetime), former smokers (smoked >100 cigarettes in a lifetime and do not smoke now), and current smokers (smoked >100 cigarettes in a lifetime and smoke some days or every day). For physical activity, a metabolic equivalent (MET) value was assigned according to the compendium of activity energy costs for each activity in the questionnaire, and the total volume of physical activity was converted into MET-minutes per week.

The average caloric consumption across a span of two days was calculated based on responses from a dietary survey questionnaire. Outlying data points were identified and removed, defined for males as caloric intakes below 800 kcal/day or exceeding 4200 kcal/day, and for females as intakes below 500 kcal/day or surpassing 3500 kcal/day. The full measurement technique for these variables is available at https://www.cdc.gov/nchs/nhanes/.

### 2.3 Statistical analyses

Statistical analyses were conducted according to the Centers for Disease Control and Prevention guidelines for analysis of NHANES data (https://wwwn.cdc.gov/nchs/nhanes/tutorials/default.aspx). A full sample 10-year mobile examination centers weight was used to calculate the US non-institutionalized population.

Continuous variables were expressed as weighted means ± standard deviation or median with interquartile range, and comparisons were conducted using weighted linear regression analysis. Categorical variables were described using unweighted frequencies (weighted percentages) and were compared using the chi-squared test. These means and frequencies can be generalized to the US adult population. A multivariate linear regression model was used to study the association between the anthropometric indices and CKD. Anthropometric indices were categorized into quartiles, and trend *p* values were calculated. Tests for trend were performed by assigning scores to the quartiles of anthropometric indices and then using regression analysis to determine if there was a significant linear relationship between these scores and the outcome variable. Four models were utilized in this study. Model 1 was a crude model without adjustment for potential confounding factors. Model 2 was adjusted for demographic characteristics, including age, gender, and race. Model 3 further adjusted for lifestyle factors, such as alcohol consumption, smoking status, physical activity, and calorie intake. To ensure the robustness of our findings, several sensitivity analyses were conducted. Restricted cubic spline (RCS) regression with four knots at the 5th, 35th, 65th, and 95th percentiles of the anthropometric indices values was employed to explore dose–response associations between anthropometric indices and CKD. Subgroup and tests for interaction were performed to investigate potential interactions of covariates. These stratification factors were also considered as pre-specified potential effect modifiers. An interaction term was included to test for heterogeneity in the associations between subgroups. The interaction was tested by incorporating interaction terms into the regression model and comparing models with and without these terms using a likelihood ratio test to evaluate their significance. Ultimately, ROC curve analysis was employed to evaluate the discriminatory ability of each anthropometric measure to identify CKD. All analyses were conducted using R software version 4.2.2 (https://www.R-project.org; R Foundation, Austria). Appropriate examination weights were applied to represent the complex survey design (https://wwwn.cdc.gov/nchs/nhanes/tutorials/weighting.aspx). Statistical significance was set at *p* < 0.05

## 3. Results

### 3.1 Participant characteristics

A total of 24,162 participants were included in our study with a mean age of 47.54 and 49.1% being male. Table 1 presents the weighted baseline characteristics of the study participants, categorized based on the presence or absence of CKD. The CKD group tended to be shorter and have significantly higher values of anthropometric indicators including weight, WC, BMI, ABSI, BRI, WHtR and C-index. Moreover, the CKD group demonstrated a higher prevalence of hypertension, diabetes, CVD, and cancer. Interestingly, concerning blood lipids, the CKD group displayed higher levels of TG but lower levels of LDL, while TC and HDL were not statistically significant.

**Table 1:** Baseline characteristics of participants classified by CKD, weighted.

Characteristics^†^	Overall (*n* = 24,162)	Non-CKD (*n* = 20,028)	CKD (*n* = 4,134)	*p*- value
Age, years	47.54±16.76	45.48±15.77	60.39±17.01	<0.001*
Male, *n* (%)	11877 (49.1)	9888 (49.9)	1989 (43.9)	<0.001*
Race, *n* (%)				
Mexican American	3,614 (8.7)	3,075 (8.9)	539 (7.5)	
Other Hispanic	2,565 (6.1)	2,197 (6.3)	368 (5.0)	
Non-Hispanic white	9,643 (66.2)	7,817 (66.0)	1,826 (67.0)	<0.001*
Non-Hispanic Black	4,987 (10.6)	4,050 (10.3)	937 (12.1)	
Others	3,353 (8.4)	2,889 (8.5)	464 (7.7)	
Marital status, n (%)				
Married	12,405 (55.2)	10,346 (55.6)	2,059 (53.0)	
Widowed	1,734 (5.3)	1,028 (3.8)	706 (18.4)	
Divorced	2,651 (10.3)	2,109 (9.9)	542 (12.7)	<0.001*
Separated	832 (2.4)	688 (2.4)	144 (2.6)	
Never married	4,514 (18.4)	4,040 (19,5)	474 (11.1)	
Living with partner	2,026 (8.4)	1,817 (8.8)	209 (5.9)	
Education level, n (%)				
Less than high school	2,382 (5.1)	1,806 (4.6)	576 (8.0)	
High school	8,587 (32.2)	6,965 (31.4)	1,622 (37.3)	<0.001*
More than high school	13,193 (62.7)	11,257 (64.0)	1,936 (54.8)	
Household income, n (%)				
0–130% FPL	7,687 (21.4)	6,250 (21.0)	1,434 (25.0)	
>130–350% FPL	9,044 (35.5)	7,350 (34.6)	1,694 (40.4)	<0.001*
>350% FPL	7,431 (43.0)	6,428 (44.4)	1,003 (34.6)	
CVD, n (%)	1,924 (6.6)	1,062 (4.6)	862(18.6)	<0.001*
Hypertension, n (%)	8,662 (31,8)	6,035 (27.3)	2,627 (59.9)	<0.001*
Diabetes, n (%)	4,567 (14.2)	2,861 (10.8)	1,706 (35.4)	<0.001*
Cancer, n (%)	2,217 (10.2)	1,507 (8.8)	710 (19.0)	<0.001*
Smoking, n (%)				
No	13,664 (56.2)	11,547 (56.9)	2,117 (51.3)	
Former	5,680 (24.7)	4,355 (23.3)	1,325 (33.3)	<0.001*
Current	4,818 (19.1)	4,126 (19.7)	692 (15.4)	
Drinking, n (%)				
No	7,274 (23.6)	5,764 (22.3)	1,528 (31.9)	
Low-to-moderate	14,926 (66.8)	12,642 (68.1)	2,284 (58.6)	<0.001*
Heavy	1.962 (9.7)	2,284 (9.7)	322 (9.5)	
MET, min/week				
<600	9381 (34.3)	7185 (31.8)	2196 (49.3)	
600–3999	8722 (38.8)	7477 (39.8)	1245 (32.5)	<0.001*
≥4000	6059 (26.9)	5366 (28.3)	693 (18.3)	
Calorie, kcal/day	2048.98±710.79	2074.69±713.21	1888.72±673.81	<0.001*
Hemoglobin, g/L	14.211.44	14.27±1.40	13.81±1.62	<0.001*
Albumin, g/L	43.0 (41.00, 45.00)	43.0 (41.00, 45.00)	42.0 (40.00, 44.00)	<0.001*
TC, mmol/L	4.98±1.06	4.98±1.05	4.96±1.17	0.528
TG, mmol/L	1.12 (0.77, 1.68)	1.09 (0.76, 1.65)	1.28 (0.87, 1.87)	<0.001*
HDL, mmol/L	1.39±0.43	1.39±0.42	1.37±0.47	0.074
LDL, mmol/L	2.96±0.92	2.97±0.90	2.89±0.99	0.001*
HbA1c, %	5.64±0.94	5.55±0.79	6.20±1.47	<0.001*
Serum creatinine, μmol/L	75.14 (63.65, 87.52)	74.26 (62.76, 84.86)	89.28 (69.84, 111.38)	<0.001*
Urea nitrogen, mmol/L	4.64 (3.57, 5.71)	4.64 (3.57, 5.71)	5.71 (4.28, 7.85)	<0.001*
eGFR, ml/min/1.73m^2^	94.38±21.62	97.72±18.08	73.54±29.01	0.082
UACR, mg/g	6.60 (4.39, 11.64)	6.03 (4.18, 9.33)	41.37 (12.81, 93.02	<0.001
Low eGFR, *n* (%)	1,956 (6.5)	-	1,956 (46.9)	<0.001*
Proteinuria, *n* (%)	2,852 (9.2)	-	2,852 (66.3)	<0.001*
Height, kg	167.00 ± 10.15	167.35 ± 10.14	165.28 ± 10.06	<0.001
Weight, cm	81.71 ± 21.42	81.48 ± 21.12	82.82 ± 22.77	<0.001
BMI, kg/m2	29.11 ± 6.80	28.91 ± 6.68	30.32 ± 7.39	<0.001*
WC, cm	99.57 ± 16.53	98.84 ± 16.23	104.16 ± 17.61	<0.001*
ABSI	0.082 ± 0.005	0.081 ± 0.005	0.084 ± 0.005	<0.001*
BRI	5.00 (3.77, 6.61)	4.88 (3.68, 6.42)	5.93(4.49, 7.69)	<0.001*
WHtR	0.60±0.10	0.59±0.10	0.63 (0.10)	<0.001*
C-index	1.31±0.09	1.30±0.09	1.35±0.08	<0.001*

CKD, chronic kidney disease; FPL, family income to poverty; CVD, cardiovascular disease; BMI, body mass index; WC, waist circumference; ASBI, a body shape index; BRI, body roundness index; WHtR, Waist-to-Height Ratio, C-index, the Conicity Index, MET, metabolic equivalent; TC, total cholesterol; TG, triglyceride; eGFR, Estimated glomerular filtration rate; UACR, urine albumin/creatinine ratio. * *p* < 0.05, ^†^ *n* (%): Unweighted numbers (weighted percentage).

### 3.2 Associations of anthropometric indices with kidney function

**Fig 2:**
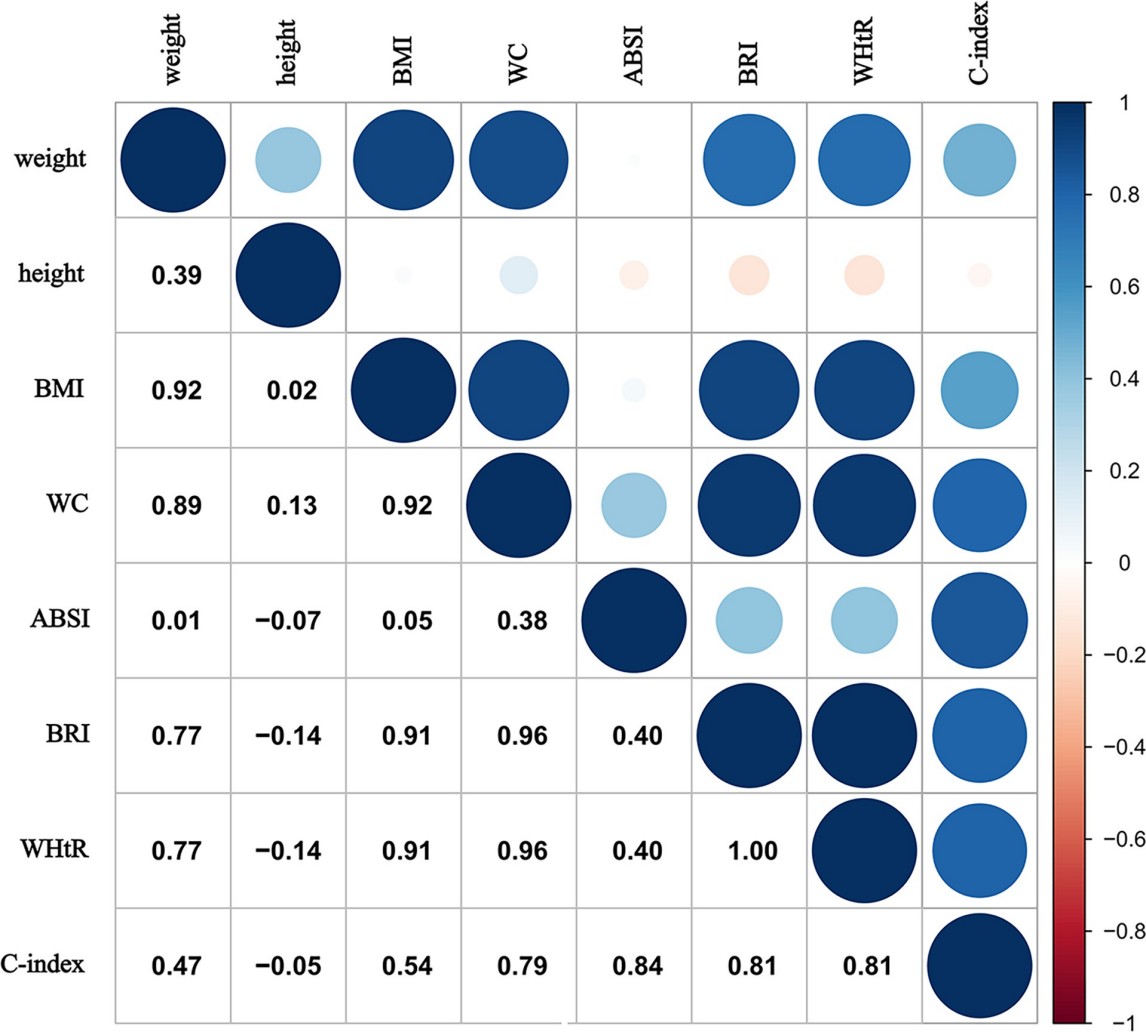
Pairwise Pearson correlations among anthropometric indices. Anthropometric indices were sex-specific z-scores transformed. BMI, Body Mass Index; WC, waist circumference; ABSI, A Body Shape Index; BRI, Body Roundness Index; WHtR, Waist-to-Height Ratio; C-index, Conicity Index.

To highlight the relationships between various anthropometric indices, the Pearson correlation was employed (Fig 2). The most notable correlations are observed between BMI and WC (r = 0.92), as well as between WC and BRI (r = 0.96), indicating strong positive associations. In contrast, ABSI shows weak correlations with most other indices, particularly with BMI (r = 0.05) and WC (r = 0.38), suggesting that ABSI captures distinct aspects of body composition. The C-index exhibits moderate correlations with BMI, WC, BRI, and WHtR, underscoring its potential utility in assessing health risks associated with obesity.

Table 2 presents the results of logistic regression analyses assessing the associations between CKD and sex-specific z-scores of various anthropometric indices. In Model 3, after adjusting for various covariates, height shows a protective effect in CKD with an OR of 0.91 (95% CI: 0.85–0.98, *p* = 0.014). Conversely, ABSI, BRI, WHtR, and C-index exhibited an increased risk of prevalent CKD, with ORs of 1.17 (95% CI: 1.11–1.23, *p* < 0.001), 1.30 (95% CI: 1.23–1.38, *p* < 0.001), 1.31 (95% CI: 1.22–1.38, *p*< 0.001), and 1.29 (95% CI: 1.22–1.36, *p* < 0.001) respectively. Additionally, significant positive associations with CKD were also observed for WC, weight, and BMI.

**Table 2:** Logistics regression for the associations between CKD and sex-specific z-scores of anthropometric indices, weighted.

	Model 1		Model 2		Model 3	
	*OR* (95% CI)	*p*-value	*OR* (95% CI)	*p*-value	*OR* (95% CI)	*p*-value
WC	1.38 (1.32, 1.44)	<0.001*	1.28 (1.20, 1.36)	<0.001*	1.27 (1.20, 1.35)	<0.001*
height	0.74 (0.69, 0.79)	<0.001*	0.89 (0.83, 0.96)	0.003*	0.91 (0.85, 0.98)	0.014*
weight	1.10 (1.05, 1.15)	<0.001*	1.20 (1.13, 1.28)	<0.001*	1.21 (1.13, 1.29)	<0.001*
BMI	1.22 (1.16, 1.27)	<0.001*	1.25 (1.18, 1.33)	<0.001*	1.25 (1.18, 1.33)	<0.001*
ABSI	1.80 (1.72, 1.88)	<0.001*	1.19 (1.13, 1.25)	<0.001*	1.17 (1.11, 1.23)	<0.001*
BRI	1.45 (1.39, 1.51)	<0.001*	1.32 (1.24, 1.39)	<0.001*	1.30 (1.23, 1.38)	<0.001*
WHtR	1.49 (1.42, 1.55)	<0.001*	1.32 (1.24, 1.40)	<0.001*	1.30 (1.23, 1.38)	<0.001*
C-index	1.80 (1.72, 1.88)	<0.001*	1.32 (1.24, 1.39)	<0.001*	1.29 (1.22, 1.36)	<0.001*

CKD, chronic kidney disease.

* *p* < 0.05; OR: weighted odds ratio. Model 1 was crude model. Model 2 was adjusted for age + sex + race, Model 3 was adjusted for Model 2 + smoking status + drinking status + Physical activity + Calorie intake.

Further dividing the anthropometric indices into quartiles, we obtained similar results (Table 3). In Model 3, the OR for Q4 of height compared to Q1 is 0.73 (95% CI: 0.56–0.96, *p* = 0.033), suggesting a significant protective effect. For ABSI, BRI, WHtR, and C-index, the odds ratios for Q4 compared to Q1 all show significant associations with increased CKD risk, with ORs of 1.37, 1.81, 1.84, and 1.79, respectively. The odds ratios for Q4 compared to Q1 for WC, weight, and BMI show a significant protective association, while Q2 and Q3 compared to Q1 did not demonstrate significance.

**Table 3:** Logistics regression for the associations between CKD and quantiles of anthropometric indices, weighted.

	Model 1		Model 2		Model 3	
	*OR* (95% CI)	*p*-value	*OR* (95% CI)	*p*-value	*OR* (95% CI)	*p*-value
Classified by WC quantiles						
Q1	1.0	-	1.0	-	1.0	-
Q2	1.18 (1.01, 1.38)	0.049*	0.87 (0.73, 1.03)	0.109	0.86 (0.73, 1.03)	0.101
Q3	1.66 (1.42, 1.94)	<0.001*	1.09 (0.92, 1.28)	0.335	1.07 (0.90, 1.26)	0.435
Q4	2.30 (2.06, 2.57)	<0.001*	1.63 (1.41, 1.88)	<0.001*	1.59 (1.38, 1.84)	<0.001*
*p* for trend		<0.001*		<0.001*		<0.001*
Classified by height quantiles						
Q1	1.0	-	1.0	-	1.0	-
Q2	0.71 (0.60, 0.83)	<0.001*	0.90 (0.74, 1.09)	0.285	0.93 (0.76, 1.13)	0.447
Q3	0.63 (0.54, 0.72)	<0.001*	0.85 (0.69, 1.06)	0.154	0.88 (0.71, 1.10)	0.259
Q4	0.46 (0.39, 0.54)	<0.001*	0.69 (0.53, 0.90)	0.007*	0.73 (0.56, 0.96)	0.025*
*p* for trend		<0.001*		0.011*		0.033*
Classified by weight quantiles						
Q1	1.0	-	1.0	-	1.0	-
Q2	0.88 (0.76, 1.01)	0.068	0.90 (0.76, 1.06)	0.198	0.90 (0.77, 1.06)	0.219
Q3	0.90 (0.79, 1.04)	0.149	0.97 (0.83, 1.13)	0.686	0.98 (0.84, 1.14)	0.780
Q4	1.06 (0.96, 1.18)	0.249	1.36 (1.17, 1.58)	<0.001*	1.36 (1.16, 1.59)	<0.001*
*p* for trend		0.166		<0.001*		<0.001*
Classified by BMI quantiles						
Q1	1.0	-	1.0	-	1.0	-
Q2	1.13 (0.97, 1.31)	0.128	0.93 (0.79, 1.10)	0.400	0.92 (0.78, 1.09)	0.323
Q3	1.23 (1.09, 1.40)	0.001*	1.01 (0.87, 1.16)	0.915	1.01 (0.86, 1.16)	0.994
Q4	1.70 (1.50, 1.92)	<0.001*	1.57 (1.35, 1.82)	<0.001*	1.54 (1.32, 1.80)	<0.001*
*p* for trend		<0.001*		<0.001*		<0.001*
Classified by ABSI quantiles						
Q1	1.0	-	1.0	-	1.0	-
Q2	1.31 (1.13, 1.52)	<0.001*	0.99 (0.85, 1.15)	0.859	0.97 (0.83, 1.13)	0.709
Q3	2.15 (1.89, 2.43)	<0.001*	1.23 (1.06, 1.41)	0.006*	1.20 (1.04, 1.38)	0.015*
Q4	3.89 (3.43, 4.40)	<0.001*	1.44 (1.24, 1.68)	<0.001*	1.37 (1.18, 1.60)	<0.001*
*p* for trend		<0.001*		<0.001*		<0.001*
Classified by BRI quantiles						
Q1	1.0	-	1.0	-	1.0	-
Q2	1.50 (1.26, 1.78)	<0.001*	0.97 (0.81, 1.15)	0.707	0.96 (0.81, 1.14)	0.604
Q3	2.19 (1.89, 2.53)	<0.001*	1.21 (1.04, 1.42)	0.009*	1.19 (1.03, 1.39)	0.023
Q4	3.32 (2.91, 3.79)	<0.001*	1.87 (1.61, 2.19)	<0.001*	1.81 (1.55, 2.12)	<0.001*
*p* for trend		<0.001*		<0.001*		<0.001*
Classified by WHtR quantiles						
Q1	1.0	-	1.0	-	1.0	-
Q2	1.50 (1.26, 1.78)	<0.001*	0.97 (0.81, 1.15)	0.710	0.99 (0.81, 1.14)	0.606
Q3	2.17 (1.88, 2.52)	<0.001*	1.22 (1.05, 1.42)	0.008*	1.19 (1.03, 1.39)	0.022
Q4	3.32 (2.91, 3.79)	<0.001*	1.89 (1.61, 2.19)	<0.001*	1.84 (1.57, 2.16)	<0.001*
*p* for trend		<0.001*		<0.001*		<0.001*
Classified by C-index quantiles						
Q1	1.0	-	1.0	-	1.0	-
Q2	1.49 (1.23, 1.79)	<0.001*	1.01 (0.83, 1.21)	0.969	1.01 (0.83, 1.21)	0.989
Q3	2.33 (1.99, 2.74)	<0.001*	1.23 (1.03, 1.45)	0.020*	1.20 (1.01, 1.42)	0.036
Q4	4.40 (3.84, 5.03)	<0.001*	1.86 (1.61, 2.15)	<0.001*	1.79 (1.54, 2.07)	<0.001*
*p* for trend		<0.001*		<0.001*		<0.001*

CKD, chronic kidney disease; eGFR, estimated glomerular filtration rate; UACR, urine albumin/creatinine ratio.

* *p* < 0.05; OR: weighted odds ratio. Model 1 was crude model. Model 2 was adjusted for age + sex + race, Model 3 was adjusted for Model 2 + smoking status + drinking status + Physical activity + Calorie intake.

Tables 4 and 5 present the results of logistic regression analyses evaluating the associations between decreased kidney function (eGFR < 90 mL/min/1.73 m^2^) and proteinuria (UACR ≥ 30 mg/g) with sex-specific z-scores of various anthropometric indices. In Model 3, WC, weight, BMI, BRI, WHtR, and C-index remained significantly associated with decreased kidney function, while height and ABSI did not show significant associations. Similarly, in Model 3 for proteinuria, all anthropometric indices were significantly associated. Most indices, including WC, weight, BMI, ABSI, BRI, WHtR, and C-index, were positively associated with proteinuria, with the exception of height, which demonstrated a protective association with an odds ratio of 0.88.

**Table 4:** Logistics regression analysis for the associations between decreased kidney function (eGFR < 90 mL/min/1.73 m^2^) and sex-specific z-scores of anthropometric indices, weighted.

	Model 1		Model 2		Model 3	
	OR (95% CI)	*p*-value	OR (95% CI)	*p*-value	OR (95% CI)	*p*-value
WC	1.38 (1.31, 1.45)	<0.001*	1.32 (1.22, 1.43)	<0.001*	1.30 (1.19, 1.41)	<0.001*
height	0.70 (0.64, 0.75)	<0.001*	1.01 (0.91, 1.11)	0.854	1.05 (0.95, 1.15)	0.367
weight	1.01 (0.94, 1.07)	0.959	1.29 (1.19, 1.41)	<0.001*	1.28 (1.17, 1.41)	<0.001*
BMI	1.12 (1.07, 1.18)	<0.001*	1.29 (1.19, 1.39)	<0.001*	1.26 (1.16, 1.38)	<0.001*
ABSI	2.28 (2.11, 2.47)	<0.001*	1.08 (0.99, 1.18)	0.072	1.07 (0.98, 1.18)	0.126
BRI	1.43 (1.36, 1.49)	<0.001*	1.29 (1.20, 1.39)	<0.001*	1.26 (1.17, 1.36)	<0.001*
WHtR	1.50 (1.43, 1.57)	<0.001*	1.31 (1.22, 1.42)	<0.001*	1.28 (1.18, 1.39)	<0.001*
C-index	2.13 (1.99, 2.28)	<0.001*	1.26 (1.16, 1.37)	<0.001*	1.23 (1.13, 1.35)	<0.001*

eGFR, estimated glomerular filtration rate.

* *p* < 0.05. Model 1 was crude model. Model 2 was adjusted for age + sex + race, Model 3 was adjusted for Model 2 + smoking status + drinking status + Physical activity + Calorie intake.

**Table 5:** Logistics regression analysis for the associations between proteinuria (UACR ≥ 30 mg/g) and sex-specific z-scores of anthropometric indices, weighted.

	Model 1		Model 2		Model 3	
	*OR* (95% CI)	*p*-value	OR (95% CI)	*p*-value	OR (95% CI)	*p*-value
WC	1.35 (1.28, 1.42)	<0.001*	1.29 (1.21, 1.37)	<0.001*	1.28 (1.20, 1.36)	<0.001*
height	0.75(0.71, 0.80)	<0.001*	0.86 (0.81, 0.93)	<0.001*	0.88 (0.82, 0.94)	<0.001*
weight	1.13 (1.07, 1.20)	<0.001*	1.19 (1.12, 1.27)	<0.001*	1.19 (1.12, 1.27)	<0.001*
BMI	1.24 (1.18, 1.31)	<0.001*	1.25 (1.17, 1.33)	<0.001*	1.25 (1.17, 1.33)	<0.001*
ABSI	1.53 (1.46, 1.61)	<0.001*	1.27 (1.20, 1.35)	<0.001*	1.24 (1.17, 1.31)	<0.001*
BRI	1.42 (1.35, 1.50)	<0.001*	1.33 (1.25, 1.42)	<0.001*	1.32 (1.25, 1.41)	<0.001*
WHtR	1.44 (1.36, 1.52)	<0.001*	1.34 (1.25, 1.43)	<0.001*	1.32 (1.24, 1.41)	<0.001*
C-index	1.59 (1.51, 1.68)	<0.001*	1.37 (1.29, 1.46)	<0.001*	1.34 (1.26, 1.43)	<0.001*

UACR, urine albumin/creatinine ratio.

* *p* < 0.05. Model 1 was crude model. Model 2 was adjusted for age + sex + race, Model 3 was adjusted for Model 2 + smoking status + drinking status + Physical activity + Calorie intake.

### 3.3. The dose-response association between anthropometric indices and CKD

**Fig 3:**
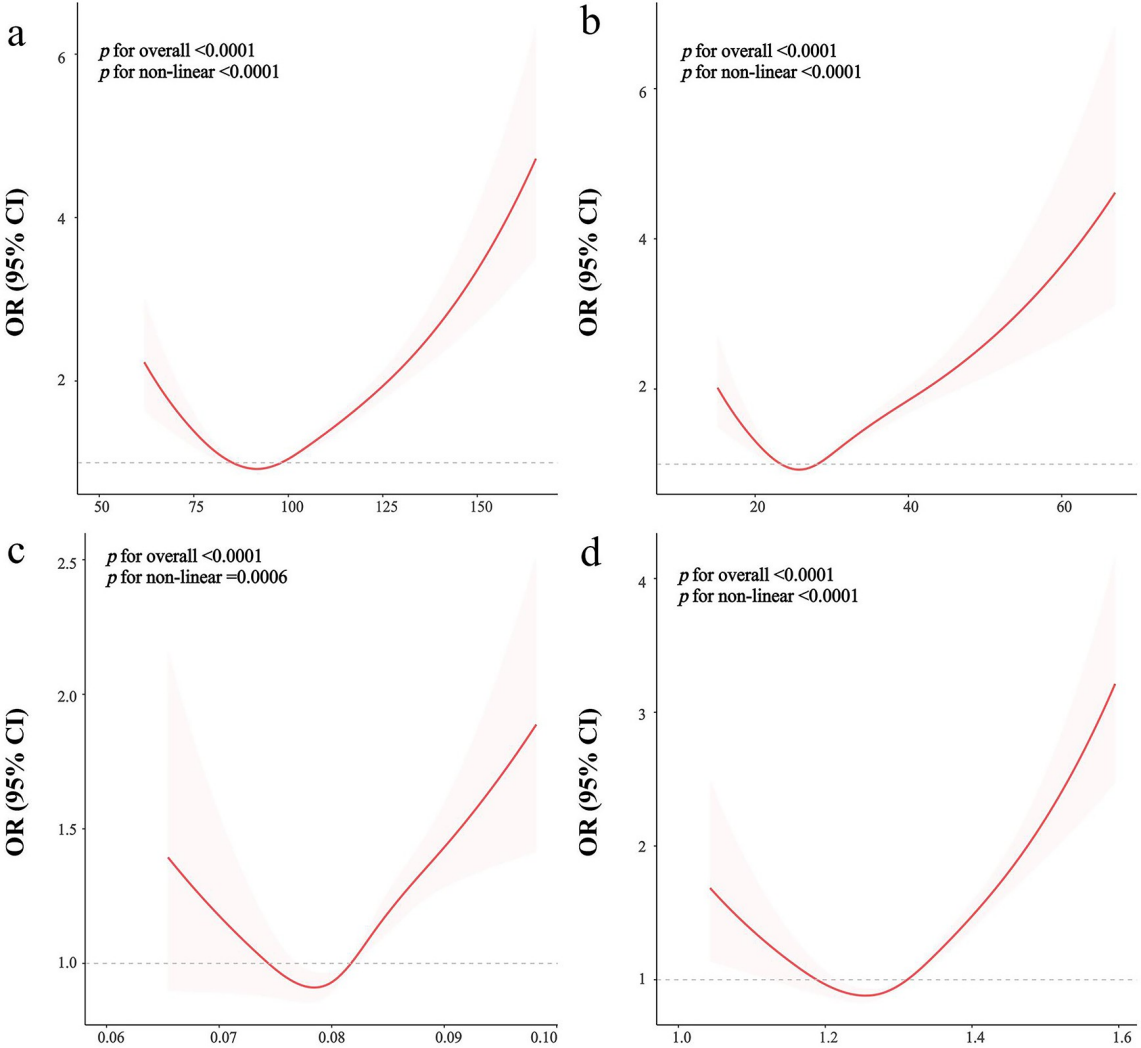
The dose–response association between anthropometric and CKD. The dose-response relationship was evaluated using restricted cubic spline regression for WC(a), BMI (b), ABSI (c), and C-index (d) and CKD, with covariates adjusted as in model 3. The odds ratio is represented by the red line and the 95% confidence interval is shown in pink.

To examine the dose-response association between the anthropometric indices and prevalent CKD, the RCS regression was performed. Fig 3 shows non-linear associations between WC, BMI, ABSI, and C-index levels and CKD (*p* for non-linear < 0.05) after adjusting for covariates. S1 Fig also demonstrated that weight, BRI, and WHtR exhibit significant nonlinear associations with CKD risk (*p* for non-linear < 0.001), whereas height maintains a linear relationship (*p* for non-linear = 0.729).

### 3.4 Subgroup analysis

**Fig 4:**
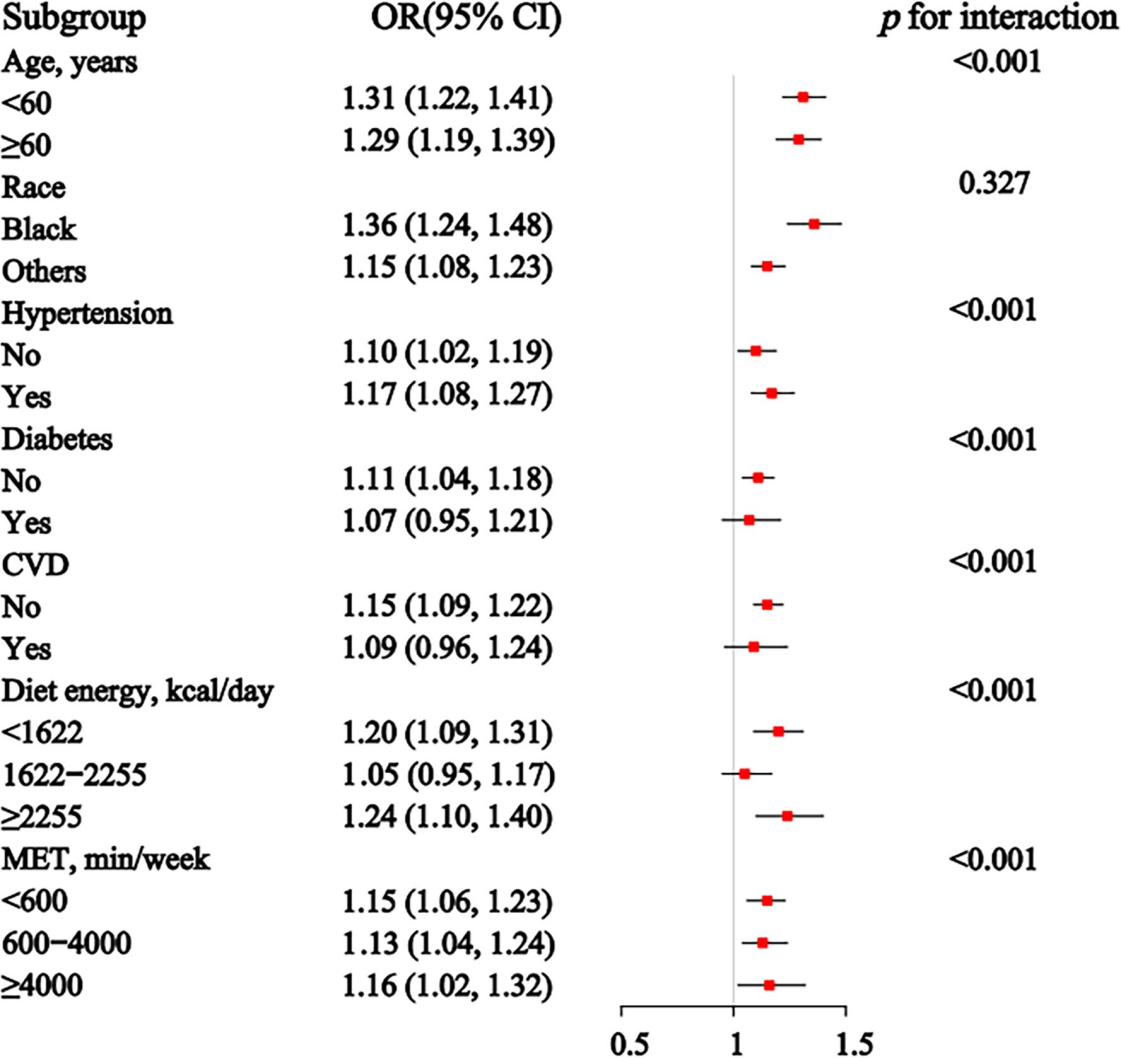
Association between the sex-specific z-score of ABSI and CKD in subgroup and tests for interaction, with covariates adjusted as in model 3. * *p* < 0.05. The number of CKD cases per category for categorical variables is as follows: for age, there are 1448 cases in the <60 group and 2686 cases in the ≥60 group; for race, there are 937 cases among Black individuals and 3197 cases among others; for hypertension, there are 1507 cases with no hypertension and 2627 cases with hypertension; for diabetes mellitus, there are 2427 cases without diabetes and 1707 cases with diabetes; for cardiovascular disease, there are 3068 cases without cardiovascular disease and 1066 cases with cardiovascular disease. For continuous variables, the number of CKD cases per group is as follows: for diet energy, there are 1755 cases in the <1622 kcal/day group, 1346 cases in the 1622–2255 kcal/day group, and 1033 cases in the ≥2255 kcal/day group; for MET, there are 2194 cases in the <600 min/week group, 1245 cases in the 600–4000 min/week group, and 695 cases in the ≥4000 min/week group.

In the subgroup analysis (Fig 4), the association between sex-specific z-scores of ABSI and CKD remained significant across all age groups, races, with or without hypertension, without diabetes or CVD, and for individuals with daily dietary energy intake below 1622 kcal or above 2255 kcal, as well as across all physical activity levels. The *p*-values for interaction indicate significant interactions for age, disease status (hypertension, diabetes, and CVD), dietary energy intake, and physical activity levels, suggesting that these factors modify the relationship between ABSI and CKD.

**Fig 5:**
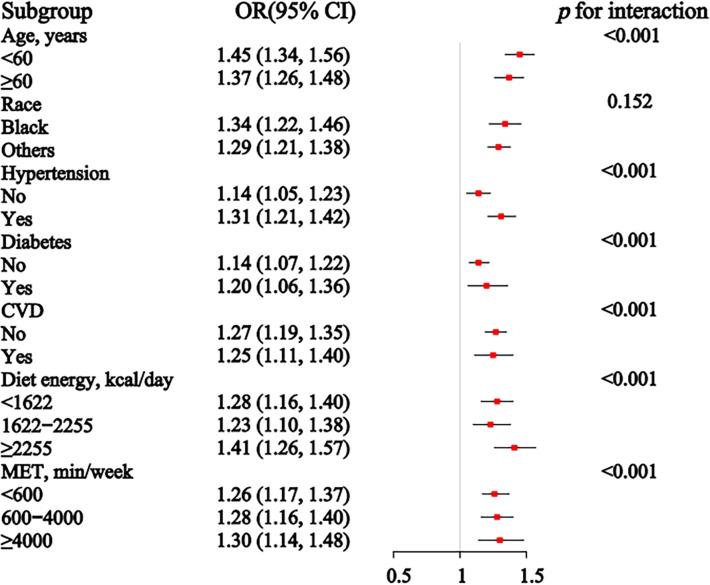
Association between the sex-specific z-score of C-index and CKD in subgroup and tests for interaction, with covariates adjusted as in model 3. * *p* <0.05.

The subgroup analysis of the associations between sex-specific z-scores of the C-index and CKD in Fig 5 demonstrates that the relationship between the C-index and CKD remains significant across all subgroups. The interaction *p*-values highlight significant interactions with age, disease conditions (including hypertension, diabetes, and CVD), dietary energy intake, and levels of physical activity, indicating that these factors significantly influence the relationship between the C-index and CKD risk.

### 3.5 ROC analyses of anthropometric indices in relation to CKD risk

**Fig 6:**
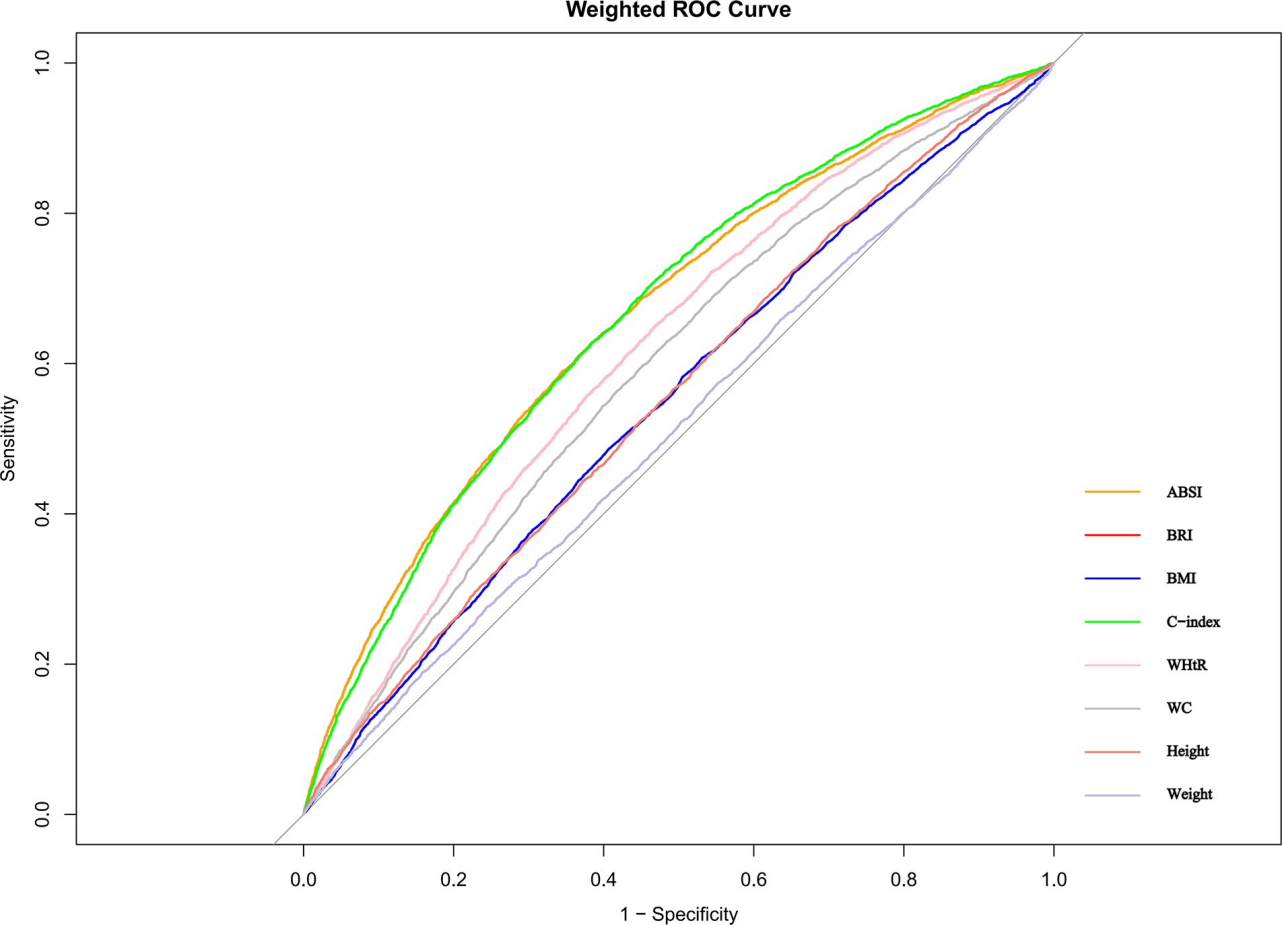
ROC curves for the prediction of CKD by anthropometric indices, weighted.

The ROC analysis results presented in Table 6 and Fig 6 indicate the predictive performance of various anthropometric indices for CKD. Among the indices, ABSI and the C-index demonstrate the highest areas under the curve (AUCs), with values of 0.655 (95% CI: 0.644–0.673) and 0.657 (95% CI: 0.665–0.674), respectively. These values suggest that ABSI and the C-index are the most effective predictors of CKD compared to the other anthropometric measures analyzed. BRI and WHtR also show relatively high AUCs of 0.610 and 0.609, respectively, indicating moderate predictive capability. Conversely, traditional measures such as WC, Height, Weight, and BMI have lower AUCs, ranging from 0.504 to 0.586, suggesting limited predictive value for CKD in this cohort.

**Table 6:** Area under the ROC curve (AUC) with 95% confidence intervals for the prediction of CKD by anthropometric indices.

Anthropometric indices	AUC	95% CI
WC	0.586	0.595–0.605
Height	0.546	0.555–0.565
Weight	0.504	0.514–0.524
BMI	0.541	0.550–0.560
ABSI	0.655	0.664–0.673
BRI	0.610	0.619–0.628
WHtR	0.609	0.618–0.628
C-index	0.657	0.665–0.674

## 4. Discussion

In this study, we explored the association between different anthropometric indices, and the risk of developing CKD. After adjusting for multiple covariates in the adult population using NHANES data from 2009 to 2018, we identified a positive correlation between ABSI, BRI, WHtR, and the C-index with CKD. However, BMI was not significantly associated with the risk of CKD. This can be attributed to the inclusion of WC in these indices, providing a more accurate reflection of obesity and fat distribution, particularly visceral fat, which plays a crucial role in CKD pathogenesis.

ABSI and the C-index demonstrated superior predictive capabilities for CKD risk in our study, as evidenced by the highest AUC values. These findings suggest that ABSI and the C-index could serve as practical and cost-effective tools for identifying CKD patients, particularly those with uneven fat distribution.

Other literature supports the superior predictive performance of ABSI over BMI in various diseases. Unfortunately, there are still relatively few studies comparing the C index with BMI. For example, a study involving 62,514 Japanese urban residents found that ABSI and the C-index had the strongest correlation with arterial stiffness among different anthropometric indices (including BMI, WC, ABSI, C-index, WHtR, and WC/BMI ratio) [27]. Another 13-year follow-up study showed that ABSI had a stronger association with increased mortality and frailty compared to BMI [28]. In men with prostate cancer diagnosed within 4 years, high ABSI, but not BMI, was independently associated with increased prostate cancer-specific mortality [29].

ABSI, which accounts for WC, height, and weight, provides a more nuanced assessment of body fat distribution and health risk compared to BMI, which is based solely on height and weight. Some studies also found that ABSI correlated minimally with BMI [27, 28, 30]. The minimal correlation between ABSI and BMI indicates that they measure different aspects of body composition and health risks. Therefore, while BMI gives a general idea of body mass relative to height, ABSI offers more specific insights into abdominal fat distribution and related health risks. Some studies have combined ABSI and BMI, achieving better results. For instance, BMI combined with ABSI has been shown to best identify obesity-related non-alcoholic fatty liver disease (NAFLD) risk, performing significantly better than BMI, WC, or ABSI alone [31].

However, not all studies agree on the superiority of ABSI or the C-index. For certain diseases, BMI performs better. For example, a meta-analysis found that BRI was superior to ABSI and similar to BMI in predicting hypertension [32]. A rural Chinese cohort study found that BMI was superior to the C-index for predicting incident hypertension [33]. Additionally, a 24-year follow-up study found that BMI was a better marker than the C-index for predicting coronary heart disease (CHD) incidence and mortality [34].

Our study also conducted subgroup analyses and tests for interaction to explore potential interactions between anthropometric indices and various diseases (hypertension, diabetes mellitus (DM), and cardiovascular disease (CVD)). Significant interactions were found, consistent with existing studies that report associations between obesity and these diseases [35–43]. Excessive adiposity is known to raise blood pressure and accounts for 65–75% of primary hypertension [35]. Obesity significantly drives DM, affecting nearly 10.5% of the global population [39], and has been correlated with increased cardiovascular and all-cause mortality over a 40-year follow-up period [36].

This study has several limitations. Firstly, its cross-sectional design limits the ability to establish or confirm causality. Secondly, despite adjusting for potential confounding factors, residual confounding factors may still influence the relationship between ABSI (or BRI) and CKD. Thirdly, the study population comprised Americans aged over 20, excluding specific groups like pregnant individuals. The limited sample size also precluded the analysis of special populations or other ethnicities. Future research should aim to ascertain the generalizability of the associations between ABSI and C-index with CKD across diverse populations. In conclusion, our findings underscore the importance of considering advanced anthropometric indices like ABSI and the C-index in CKD risk assessment. These indices provide valuable insights into body fat distribution, particularly visceral fat, which is crucial in understanding and managing CKD risk.

### 5. Conclusion

This study highlights the significant associations between various anthropometric indices and the risk of CKD. Height was found to have a protective effect against CKD. Among the indices studied, ABSI and the C-index demonstrated the strongest predictive capabilities for CKD, with the highest AUC values. Conversely, traditional measures such as BMI, weight and WC showed limited predictive value. The findings underscore the importance of using comprehensive anthropometric assessments in identifying and managing CKD risk, with ABSI and the C-index emerging as valuable tools in this context. Further research is needed to confirm these associations across diverse populations and explore the underlying mechanisms.

## Supporting information

S1 Fig The dose–response association between anthropometric and CKD. The dose-response relationship was evaluated using restricted cubic spline regression for weight (a), height (b), BRI (c), and WHtR (d) and CKD, with covariates adjusted as in model 3. The odds ratio is represented by the red line and the 95% confidence interval is shown in pink.
(TIF)
