# Supplementary figures and images for: Association between anthropometric indices and chronic kidney disease: Insights from NHANES 2009–2018
Source: PLoS One. 2025 Feb 14;20(2):e0311547. doi: 10.1371/journal.pone.0311547 (PMC11828394; doi:10.1371/journal.pone.0311547)

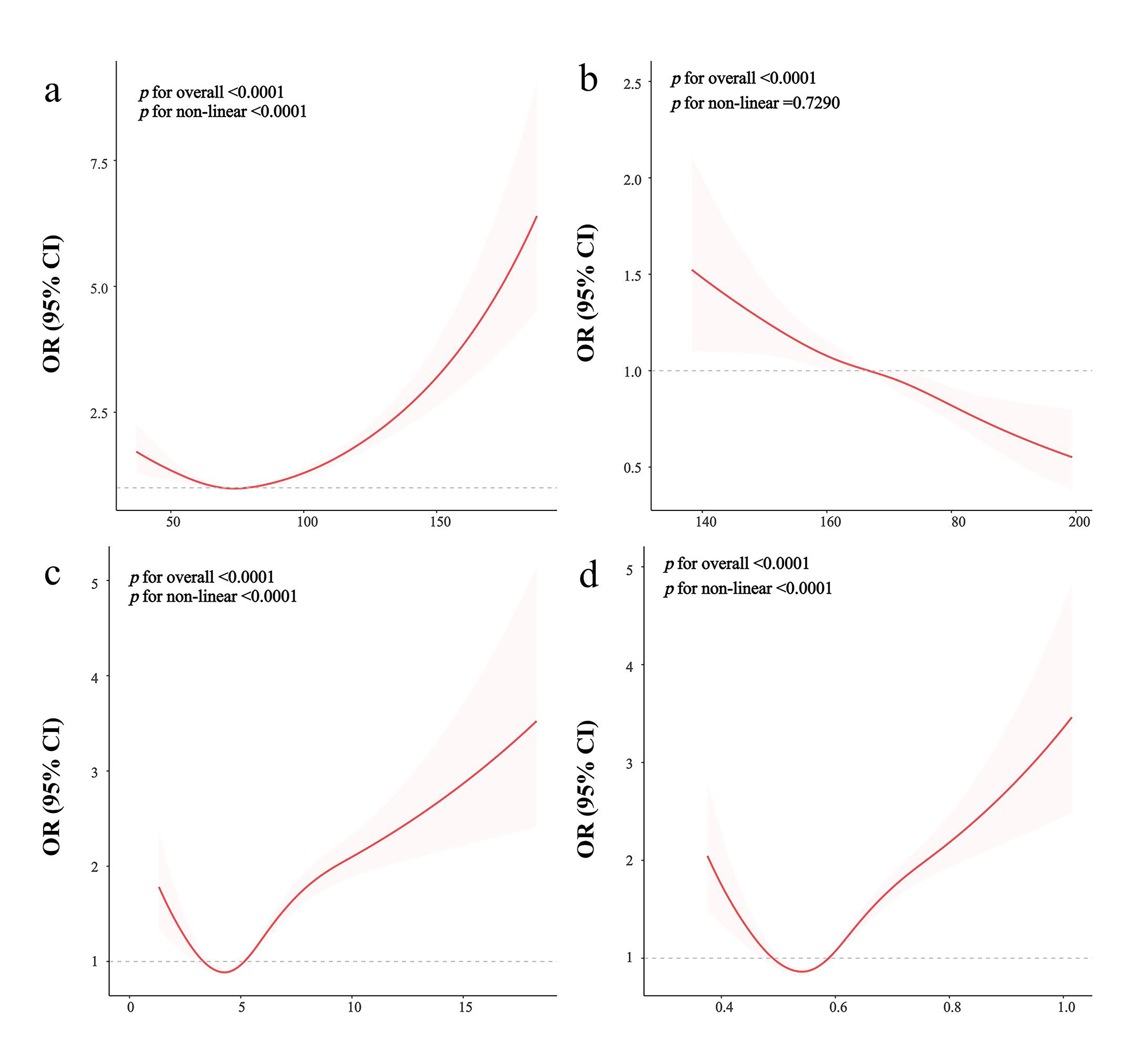

Supplement: S1 Fig — The dose-response relationship was evaluated using restricted cubic spline regression for weight (a), height (b), BRI (c), and WHtR (d) and CKD, with covariates adjusted as in model 3. The odds ratio is represented by the red line and the 95% confidence interval is shown in pink. (TIF) [file pone.0311547.s001.tif]
